# Fasciola and fasciolosis in ruminants in Europe: Identifying research needs

**DOI:** 10.1111/tbed.12682

**Published:** 2017-10-06

**Authors:** N. J. Beesley, C. Caminade, J. Charlier, R. J. Flynn, J. E. Hodgkinson, A. Martinez‐Moreno, M. Martinez‐Valladares, J. Perez, L. Rinaldi, D. J. L. Williams

**Affiliations:** ^1^ Institute of Infection and Global Health University of Liverpool Liverpool UK; ^2^ Health Protection Research Unit in Emerging and Zoonotic Infections University of Liverpool Liverpool UK; ^3^ Avia‐GIS Zoersel Belgium; ^4^ Universidad de Cordoba Cordoba Spain; ^5^ Instituto de Ganaderia de Montana CSIC Universidad de Leon Grulleros Leon Spain; ^6^ Department of Veterinary Medicine and Animal Productions University of Naples Federico II Napoli Italy

**Keywords:** diagnosis, *Fasciola hepatica*, fluke, fluke vaccine, flukicide resistance, Galba, helminth immunomodulation, research gaps, socio‐economics of parasite infection, transmission

## Abstract

*Fasciola hepatica* is a trematode parasite with a global distribution, which is responsible for considerable disease and production losses in a range of food producing species. It is also identified by WHO as a re‐emerging neglected tropical disease associated with endemic and epidemic outbreaks of disease in human populations. In Europe, *F. hepatica* is mostly associated with disease in sheep, cattle and goats. This study reviews the most recent advances in our understanding of the transmission, diagnosis, epidemiology and the economic impact of fasciolosis. We also focus on the impact of the spread of resistance to anthelmintics used to control *F. hepatica* and consider how vaccines might be developed and applied in the context of the immune‐modulation driven by the parasite. Several major research gaps are identified which, when addressed, will contribute to providing focussed and where possible, bespoke, advice for farmers on how to integrate stock management and diagnosis with vaccination and/or targeted treatment to more effectively control the parasite in the face of increasing the prevalence of infection and spread of anthelmintic resistance that are likely to be exacerbated by climate change.

## 1 INTRODUCTION

*Fasciola hepatica* is a trematode parasite found throughout Europe which affects a range of hosts, including ruminants, horses, wild animal hosts such as deer, rabbits and hares and humans. Loss of production associated with infection and overt clinical disease results in significant costs to the global farming industry, estimated at over $3 billion per year (Spithill, Smooker, & Copeman, 1999). These costs are largely unquantified at a national or regional level, whilst at a farm level, it has been reported that fluke affects milk yield, carcase composition and extends time to reach slaughter weight (Charlier, Vercruysse, Morgan, van Dijk, & Williams, 2014; Howell, Baylis, Smith, Pinchbeck, & Williams, 2015). Evidence from across Europe suggests that both the awareness and prevalence of infection has increased in particular regions of Europe, such as southern Sweden (Höglund et al., 2010). There are growing concerns about resistance to flukicides and about drug residues in meat and milk which have led to restrictions in their use and an increase in meat and milk withdrawal periods for many products (http://www.noahcompendium.co.uk). *Fasciola hepatica* also has the capacity to modulate the host's immune system, affecting susceptibility to and diagnosis of other pathogens including bovine tuberculosis (Claridge et al., 2012).

This review will focus on fasciolosis in Europe, caused by *F. hepatica* and will build on the many recent reviews of all aspects of fluke biology, to highlight new challenges in controlling the parasite and to identify gaps where more research is urgently needed (http://www.discontools.eu). The review highlights the importance of the snail intermediate host; recent developments in epidemiology of fasciolosis and the predicted impact of climate change on its prevalence and spatial distribution; what improvements in diagnosis are needed and how better to apply drugs to slow the development and spread of resistance; and finally we consider gaps in our knowledge of fluke‐driven immunomodulation and how this relates to vaccine development.

*Fasciola hepatica* has an indirect life cycle involving lymnaeid snail intermediate hosts, the principal species in Europe being *Galba truncatula*. Undifferentiated fluke eggs are passed out in the faeces of infected animals and once washed out of the faeces, the eggs start to develop, a process dependent on temperature. When a fully developed egg is given stimuli of increased light and temperature, the short‐lived miracidium is released. It requires water to swim through and once it finds a suitable lymnaeid snail, it burrows through the foot and into the body cavity. The fluke multiplies and, after about 6 weeks, cercariae are released. Again this process is temperature dependent and a snail infected with a single miracidium can produce several hundred cercariae, released over a period of time. The cercariae encyst on the vegetation to form infective metacercariae. When a grazing animal eats contaminated herbage, the metacercariae hatch, the newly excysted juveniles burrow through the gut wall and migrate into the liver. Immature fluke migrate through the liver parenchyma for 6–8 weeks before entering into bile ducts, where they mature and start producing eggs that can then be detected in faeces (reviewed in (Dalton, 1999).

## 2 SNAIL BIOLOGY

The presence of the snail intermediate host is essential to the transmission of *F. hepatica,* and knowledge of the interaction between snail and parasite is important when considering what drives parasite transmission. It is also important to understand how events in the snail influence genetic diversity of parasites in the mammalian host. To fully understand the epidemiology of *Fasciola* spp., better knowledge of snail habitats, species of snails acting as intermediate hosts, and prevalence of *F. hepatica* infection within the snail are required (Cañete, Yong, Sánchez, Wong, & Gutiérrez, 2004).

Although *G. truncatula* are usually found in semi‐aquatic habitats (Boray, 1969), including drainage furrows, slow moving streams, temporary moist areas and banks of rivers and ponds (Charlier, Soenen et al., 2014; Rondelaud, Hourdin, Vignoles, Dreyfuss, & Cabaret, 2011; Schweizer et al., 2007), they are resistant to drought and frost; so will aestivate or hibernate by burying into the mud for extensive periods (Armour, 1975; Ollerenshaw, 1959; Schweizer et al., 2007). This means that snail habitats are only readily identifiable at certain points through the year, for example in spring/summer and autumn when there are peaks in the abundance of adult and juvenile snails, respectively (Charlier, Soenen et al., 2014; Manga‐Gonzalez, Gonzalez‐Lanza, & Otero‐Merino, 1991; Relf et al., 2011). The number and size of temporary or secondary habitat vary from year to year depending on the prevailing weather conditions, and as a result alters the carrying capacity from one year to the next (Crossland, 1976). Locating snail habitats on farms is laborious and dependent on the skills of the personnel involved (Heppleston, 1972); yet detailed characterization of snail habitats is crucial to be able to predict the risk of fasciolosis at the individual farm level (Charlier et al., 2011). Using remote sensing methods, particularly soil moisture data from the new generation of Sentinel satellite systems together with other technologies, such as detection of environmental DNA, to identify suitable snail habitat on farms will improve our ability to predict when and where metacercariae may appear on pasture. The timing of high‐risk periods will vary from region to region, for example in northern Europe, large numbers of metacercariae appear in autumn, whilst in southern Europe, risk is greater in winter and spring (Caminade, Van Dijk, Baylis, & Williams, 2015). Developing methods to identify when metacercariae appear on pasture and to quantify risk, will inform grazing and drug control programmes, particularly if combined with improved diagnostic tests.

In Europe, the main snail intermediate host is *G. truncatula*, but *Lymnaea palustris*,* Omphiscola glabra*,* Radix balthica*,* Succinea* spp. and *Potamopyrgus antipodarum* are all reported as potential intermediate hosts of *F. hepatica* (Abrous, Rondelaud, Dreyfuss, & Cabaret, 1999; Caron, Martens, Lempereur, Saegerman, & Losson, 2014; Dreyfuss, Alarion, Vignoles, & Rondelaud, 2005; Jones, Williams, Dalesman, & Brophy, 2015; Novobilský, Kašný, Beran, Rondelaud, & Höglund, 2013; Relf, Good, McCarthy, & de Waal, 2009; Rondelaud et al., 2011). Infections in these other species of snail may be accidental and may not always lead to the production of cercariae (Boray, 1969, 1978), but nevertheless other species could represent additional reservoirs of infection. Accurate identification of these snails is, therefore, important. Morphological differentiation between species of the Lymnaeidae family is possible though time consuming (Bargues & Mas‐Coma, 1997) and the internal transcribed spacer two regions can be used for molecular differentiation (Bargues, Bargues, & Mas‐Coma, 2005; Bargues et al., 2001).

Some species of snail may only be susceptible to infection with *F. hepatica* as juveniles (Boray, 1978), and it is suggested that this age‐related resistance to infection is related to the maturation of the snail immune system (Dreyfuss, Abrous, & Rondelaud, 2000). Similarly, differences in susceptibility to *F. hepatica* infection within populations of snails of the same species may be related to variation in the immune response (Gutierrez, Pointier, Yong, Sanchez, & Theron, 2003; Gutiérrez et al., 2003). Further work is required to fully understand why some individual snails produce large numbers of cercariae, whilst the overall prevalence of infection in snail populations is generally low.

Infection with *F. hepatica* can be detected by observing shedding of cercariae, crushing or microscopic dissection of the snail (Kaplan, Dame, Reddy, & Courtney, 1995). These methods have poor sensitivity in the early stages of infection, and it is difficult to distinguish the intra‐molluscan stages of different species of trematodes (Caron, Rondelaud, & Losson, 2008; Heussler, Kaufmann, Strahm, Liz, & Dobbelaere, 1993; Kaplan et al., 1995). A number of molecular techniques have been used to detect *F. hepatica* infection in snails, which have generally been found to be more sensitive than microscopic methods (Caron, Lasri, & Losson, 2007; Relf et al., 2011); however, the presence of inhibitory factors within the snails can reduce the sensitivity of PCRs (Cucher, Carnevale, Prepelitchi, Labbé, & Wisnivesky‐Colli, 2006) conversely it is important to ensure that putative trematode‐specific PCR primers do not amplify snail DNA. A number of other trematode species, including those of birds and amphibians, have been isolated from *G. truncatula* including *Calicophoron daubneyi*,* Haplometra cylindracea*,* Notocotylus* spp., *Plagiorchis* spp. (Rondelaud, Vignoles, & Dreyfuss, 2015). Some of these trematodes have little or no published DNA sequence available, which makes it difficult to ensure PCRs are *F. hepatica* specific. Furthermore, few of the published *F. hepatica* PCRs have been validated for use with snails.

In Europe, the prevalence of *F. hepatica* infection in *G. truncatula* is reported to be around 5% but ranges from 0.5% to 13.5% at different times of year (Mage, Bourgne, Toullieu, Rondelaud, & Dreyfuss, 2002; Rondelaud & Dreyfuss, 1997; Rondelaud, Vignoles, & Dreyfuss, 2004; Rondelaud et al., 2015). Most of these studies show only if a snail is infected, but snails can be infected and not shed cercariae; in experimental infections the proportion of snails shown to be infected with *F. hepatica* but that did not shed cercariae ranged from 12.2% to 67.6% (Dreyfuss, Rondelaud, & Vareille‐Morel, 1999; Dreyfuss, Vignoles, Rondelaud, & Vareille‐Morel, 1999). Knowing if a snail can shed cercariae is important to understand its transmission potential (Ollerenshaw, 1971), but this is difficult to achieve. Similarly, we know very little about the optimal conditions required to stimulate shedding of cercariae from snail species other than *G. truncatula* (Dreyfuss, Vignoles et al., 1999; El‐Shazly, Nabih, Salem, & Mohamed, 2012). PCRs detect infection with *F. hepatica* irrespective of whether snails have, or could shed cercariae and it is important to highlight the difference between the presence of fluke DNA in a snail, and the role a particular species has in the epidemiology of *F. hepatica*. For example, the New Zealand mudsnail *P. antipodarum* has been reported to be an intermediate host of *F. hepatica* (Jones et al., 2015), yet it has been shown that *P. antipodarum* feeds on miracidia rather than becoming infected by them (Nansen, Frandsen, & Christensen, 1976). Furthermore, snails can be infected with more than one species of trematode (Degueurce et al., 1999; Jones et al., 2015; Rondelaud et al., 2004). One infection is usually dominant over the other (Jones, Brophy, Mitchell, & Williams, 2016; Rondelaud, Vignoles, & Dreyfuss, 2007), and therefore, co‐infection in the snail may influence transmission of *F. hepatica*, but again, this is a relatively unexplored area of research.

The relatively low pathogenic effect of *F. hepatica* on the snail intermediate host means snails can survive for months and produce cercariae over a prolonged period. Moreover, the rapid clonal expansion that occurs within the snail means the parasite can produce and shed several hundred cercariae at a time (Boray, 1978; Hodasi, 1972). The time over which snails shed is variable, and shedding is not continuous. Under experimental conditions a number of factors can influence the capacity of the snail to produce cercariae, for example the number of miracidia that infect a snail as well as the nutrition, age and size of the snail (Belfaiza, Abrous, Rondelaud, Moncef, & Dreyfuss, 2004; Dreyfuss, Vignoles et al., 1999; Kendall & Ollerenshaw, 1963; Vignoles, Rondelaud, & Dreyfuss, 2010).

We have shown that parasites with the same genotype tend to occur in the same animal giving evidence of clumped transmission and that clonal expansion occurs hence multiple genetically identical cercariae are likely to be released in a relatively small area (Beesley, Williams, Paterson, & Hodgkinson, 2017). However, there is little information on how far snails roam, or the distance cercariae travel once they exit the snail before encysting. Snails may also be infected with miracidia of more than one genotype, and shed cercariae of more than one genotype at the same time. Experimentally snails can be infected with two miracidia, four hours apart (Dar, Vignoles, Dreyfuss, & Rondelaud, 2010; Dreyfuss et al., 2000), but it is not known if this occurs naturally nor if miracidia develop at different rates, and thus the proportion of genotypes of cercariae that are released may change overtime. These factors may potentially affect the distribution of metacercariae on pasture, and thus, the genotypes of parasites the definitive host is exposed to.

### 2.1 Key questions and future directions

For many years, the role of the intermediate host in the *Fasciola* spp. life cycle has been relatively neglected, but modern molecular and genomic tools are becoming available to study events in the snail, and we can start to address how these impact on transmission and the spread of virulence and anthelmintic resistance genes within fluke populations.

## 3 EPIDEMIOLOGY

The development of *F. hepatica* eggs, larval stages and its intermediate host snails in the environment are highly dependent on geo‐climatic, ecological and anthropogenic factors such as elevation, rainfall, temperature, evapotranspiration, moisture, vegetation and soil type. As a consequence, *F. hepatica* is widespread in Europe but with an uneven spatial distribution and with great regional variations in prevalence. Recently, European maps of *F. hepatica* infection risk in dairy cattle (Ducheyne et al., 2015)and sheep (Rinaldi et al., 2015)were delivered as product of the EU GLOWORM project. Furthermore, country‐specific surveys show herd‐level prevalence ranging from 7% in Sweden to 97% in the alpine upland farms (Charlier, Vercruysse et al., 2014) and from 4.0% in southern Italy to 61.6% in Ireland in sheep farms (Rinaldi et al., 2015).

Recent research efforts in the field of *F. hepatica* epidemiology have focused on quantifying and understanding the spatial distribution of *F. hepatica*, by exploiting the correlations between environmental predictors and management factors with livestock's exposure to the liver fluke (Bennema et al., 2011; McCann, Baylis, & Williams, 2010). Recently, the predictive performance of initial statistical models using standard linear or logistic regression analysis has been outperformed by more advanced modelling approaches such as random forest and boosted regression trees reaching accuracies of >95% (Ducheyne et al., 2015; Selemetas et al., 2015). Despite the important progress in this area, the spatial distribution of *F. hepatica* in southern, central and eastern Europe, remains poorly described. Furthermore, additional research is required to improve the spatial resolution of *F. hepatica* risk maps from broad administrative or farm level to pasture level so that risk maps can support the implementation of specific management advices on drainage, grazing strategies and targeted (selective) treatments. Recent studies have explored the use of very high‐resolution satellite and drone imagery to map small water bodies and the intermediate host snails on pasture, but further research is required to make this approach operational and to develop sustainable business cases (Charlier, van der Voort, Kenyon, Skuce, & Vercruysse, 2014; Charlier, Soenen et al., 2014; De Roeck et al., 2014). Enhanced capability to monitor water bodies at high spatial resolution could be provided by recent sentinel European Space Agency (ESA) satellites, which carry a range of technologies, such as radar and multi‐spectral imaging instruments for land, ocean and atmospheric observation (https://sentinel.esa.int).

Besides the potential to improve our understanding of the detailed spatial distribution, research is required to scrutinize the temporal side of *F. hepatica* epidemiology. This can be achieved using both (i) mathematical transmission models and/or forecasting systems (Baggenstos et al., 2016) and (ii) longitudinal surveys to monitor evolution in *F. hepatica* infection over time. Forecasting systems of autumnal *F. hepatica* disease risk based on correlations of temperature and rainfall with disease outbreaks have been available for a long time in some countries (e.g., in the UK and the Netherlands (see (Ollerenshaw & Rowlands, 1959) and (Gaasenbeek, Over, Noorman, & de Leeuw, 1992)). With ongoing climate change, these systems have gained renewed interest in the evaluation of its effect of on the epidemiology of *F. hepatica*. For instance, (Caminade et al., 2015) applied climate change scenarios to an existing forecasting system showing that climate change could lead to significant increases in infection risk in most parts of Europe, with an extension of the transmission season of up to 4 months in northern Europe. However, when applied for short‐term prediction of disease occurrence, the described forecasting systems are region‐specific. They cannot as such be extrapolated to other regions because they do not explicitly capture the dependence of the life cycle of *F. hepatica* on key environmental factors (Charlier, Soenen et al., 2014). Therefore, development and validation of mechanistic mathematical models are required as well as better insights into the effects of environmental conditions on the survival of eggs, metacercariae and intermediate host snails on pasture in different geographical settings. In addition, mechanistic models are not only key to the improved prediction of *F. hepatica* disease, but also for the in silico evaluation of novel control strategies such as vaccination (Turner et al., 2016).

Complementary to predictive systems, it is important to set up surveillance systems that monitor infection status at farm level on a regular basis. Such systems can capture unexpected deviations from mathematical model predictions and indicate whether farmer management is able to cope with altered disease risk or not. Recently, Charlier et al. (2016) showed that monitoring *F. hepatica*‐specific antibody levels in bulk tank milk from a randomized sample of dairy farms allowed detection of both interannual (weather‐driven) changes as well as longer‐term trends in *F. hepatica* exposure. Munita et al. (2016) showed that such longitudinal monitoring approaches can also be an effective decision support tool because it supported evidence‐based interventions leading to year‐on‐year reductions in the study farms’ infection status for *F. hepatica* in Ireland. Such monitoring approaches should also take advantage of veterinary or hunting networks for the collection of faecal samples from non‐dairy livestock and wildlife (Mezo et al., 2013; Rinaldi et al., 2015). The latter is crucial in further elucidating the role of wildlife for the introduction and the spread of novel (fasciolicide‐resistant) isolates on a farm.

### 3.1 Key questions and future directions

Critical questions about the impact of our changing world on fluke transmission remain. Better national, regional and local forecasting systems are needed to inform farmers as parasite challenge reaches its peak and sustainable surveillance systems need to be installed to validate forecasts and monitor the progress of ongoing control efforts.

## 4 SOCIO‐ECONOMICS

Over the last decade, good progress has been made in assessing the production economic impacts of *F. hepatica* in ruminants, and these have extensively been reviewed in sheep (Rojo‐Vázquez, Meana, Valcárcel, & Martínez‐Valladares, 2012) and in cattle (Charlier, Vercruysse et al., 2014). A remaining gap is to establish the impact of *F. hepatica* on fertility parameters using randomized intervention field studies. However, the major challenge is to develop tools that are able to quantify the economic impact of *F. hepatica* at national, regional and farm level to support decision‐making by governments, animal health organizations and farmers, respectively and that can be used as efficient management tools. An example is ParaCalc® where the annual economic impact on a farm is estimated based on farm‐specific diagnostic test results and observed production impacts of *F. hepatica* (Charlier, Van der Voort, Hogeveen, & Vercruysse, 2012). However, this system uses average production estimates as inputs and thus lacks farm‐specificity, and it does not reflect the effect on the whole‐farm economic performance. This is an important gap because the farmer needs to be able to compare the impact of liver fluke control with other animal health issues or general interventions and investment opportunities. Recently, this problem was addressed by the introduction of efficiency analysis to evaluate the impact of helminth infections (van der Voort et al., 2013). Efficiency analysis studies the conversion of input(s) into output(s) and compares the current performance level of a farm with the performance level of peer farms with similar production technologies (Coelli et al., 2005). A major advantage of efficiency analysis is the possibility of linking an animal disease or its diagnosis to input allocation, which is at the core of a farmer's decision‐making process. An efficiency analysis approach was successfully developed for gastrointestinal nematodes in cattle (van der Voort, Van Meensel, Lauwers, Van Huylenbroeck, & Charlier, 2015; van der Voort et al., 2014), and should now be extended to include other major endemic pathogens including *F. hepatica*. Furthermore, linking such a system to real and actual farm data is a prerequisite for its success. A closer collaboration between the model‐makers and model‐users and the stimulation to develop concrete business cases may be the critical success‐factor for these systems to become self‐sustainable in the near future.

### 4.1 Key questions and future directions

Development of bespoke farm economic models will enable farmers to make informed choices about what control programmes to adopt for their enterprise. However, national economic models which assess the changing disease burden on farm gate, national and international meat and milk prices are also necessary to feed back into farm level efficiency analysis.

## 5 DIAGNOSIS

Traditionally, fluke infections have been diagnosed by detecting eggs in faeces (Anderson et al., 1999; Boray, 1985). However, the pre‐patent period is 8–10 weeks depending on the host species; hence, egg counts are only useful from about 8‐week post‐infection (wpi) onwards. In addition, other factors such as host age, faecal water content and the number of aliquots tested per sample, can all affect the sensitivity of the faecal egg count (FEC; reviewed by Alvarez Rojas, Jex, Gasser, and Scheerlinck (2014)). False positives may occur due to the retention of eggs in the gall bladder for at least 2 weeks after successful treatment (Flanagan et al., 2011). Coprological sedimentation methods are well established in routine diagnostic laboratories, and methods such as FLOTAC (Cringoli, Rinaldi, Maurelli, & Utzinger, 2010) and Flukefinder (Foreyt, 2001) are available.

Infection can be also confirmed at necropsy and many farmers use abattoir returns to identify if *F. hepatica* is present in their livestock (Mazeri, Sargison, Kelly, Bronsvoort, & Handel, 2016).

Many immunological techniques have been described that have higher sensitivity, reproducibility and often cost‐effectiveness. Antibody detection indirect‐enzyme‐linked immunosorbent assays (ELISA) have been developed. Their diagnostic sensitivity and specificity were reviewed recently by Alvarez Rojas et al. (2014). Most antibody‐detection ELISAs are based on excretory–secretory (E/S) products, cathepsin L proteinases (CatLs), a group of endopeptidases secreted in abundant amounts by epithelial cells of immature and adult *Fasciola* sp., or a subfraction of the E/S products called F2 antigen. Infection with *Fasciola* spp. is characterized by an increase in parasite specific IgG, which is normally detectable by 4 wpi and reaches a peak between 8 and 10 wpi (Martínez‐Pérez, Robles‐Pérez, Rojo‐Vázquez, & Martínez‐Valladares, 2014; Salimi‐Bejestani et al., 2005). Whilst antibody‐detection tests have excellent sensitivity, antibodies can remain in serum for several months following successful treatment. This makes the interpretation of a positive result difficult if the full‐treatment history is not available.

Antibodies are secreted in milk, meaning that several serum antibody tests have been adapted to use either individual or bulk tank milk samples providing automated, rapid and cheaper methods for monitoring infection status in dairy herds. Most tests show good diagnostic sensitivity and specificity and several ELISAs are commercially available for detecting the infection in both milk and serum samples (Table 1).

**Table 1:** Summary of commercially available antibody‐detection ELISA tests for diagnosis of *Fasciola hepatica* in cattle

Test	Source	Reference
Fasciolosis Verification Test	Idexx, USA	Kuerpick, Schnieder, & Strube (2013)
MM3‐Sero ELISA	BIO X Diagnostics, Belgium	Mezo, González‐Warleta, Castro‐Hermida, Muiño, & Ubeira (2010)
SVANOVIR® ELISA	Boehringer Ingelheim Svanova, Sweden	(Charlier, Duchateau, Claerebout, Williams, & Vercruysse (2007)

Antibody‐detection ELISAs are normally developed for a single‐host species due to difficulties in developing good anti‐species conjugates and typically detect single‐parasite species. Ideally, tests that cover the spectrum of pasture‐borne helminthoses, multiple assays for rapid and high‐throughput diagnosis are required. Karanikola et al. (2015) developed a bead‐based assay using fluorescence detection (xMAP® technology) for the simultaneous detection of antibodies against *F. hepatica*,* Cooperia oncophora* and *Dictyocaulus viviparus* in cattle serum samples. This platform was shown to be highly sensitive and specific in comparison with existing serological and coprological diagnostic techniques.

Monoclonal antibody‐based sandwich ELISAs have been developed for the detection of circulating antigens in the sera or faeces from infected animals. A wide range of antigens from *F. hepatica* including E/S products, tegumental components, crude extracts from adult worms and recombinant proteins (such as *F. hepatica* cathepsins, a heat shock protein and a saposin‐like protein) has been incorporated into antigen detection assays (reviewed by (Alvarez Rojas et al., 2014)).

The MM3‐COPRO test, based on the MM3 MoAb that binds to both CatL1 and CatL2 proteases (Mezo, González‐Warleta, Carro, & Ubeira, 2004), is commercialized by BIO X Diagnostics (La Jemelle, Belgium). Kajugu et al. (2012, 2015) showed that this test has a high diagnostic specificity (100%), and showed no cross reaction when tested with soluble fractions of homogenates from *Paramphistomum cervi* and *Taenia hydatigena* but also in co‐infections with paramphistome, coccidian and/or gastrointestinal nematodes. However, the sensitivity of this ELISA can sometimes be compromised by the high variability in the concentration of cathepsin proteinases in faecal samples and by differences in the between‐batch performance of peroxidase‐labelled anti‐mouse IgG polyclonal antibodies (Martínez‐Sernández, Orbegozo‐Medina, González‐Warleta, Mezo, & Ubeira, 2016). Brockwell, Spithill, Anderson, Grillo, and Sangster (2013) and Palmer, Lyon, Palmer, and Forshaw (2014) improved the sensitivity of MM3‐COPRO ELISA using a customized cut‐off for sheep and cattle whilst maintaining the specificity above 99%. However, the test had poor diagnostic sensitivity in horses (Palmer et al., 2014). Recently a new version of the coproantigen test using a streptavidin‐polymerized horseradish peroxidase conjugate was evaluated and was sufficiently sensitive to detect infection with a single fluke (Martínez‐Sernández et al., 2016).

Molecular diagnostic methods have been developed to increase the sensitivity and the specificity of conventional diagnostics. Martínez‐Pérez, Robles‐Pérez, Rojo‐Vázquez, and Martínez‐Valladares (2012) developed a nested‐PCR capable of detecting the infection in faeces of sheep as early as 2 wpi by amplifying a 423 bp fragment of the cytochrome C oxidase 1 gene. Robles‐Pérez, Martínez‐Pérez, Rojo‐Vázquez, and Martínez‐Valladares (2013) also detected the infection at 2 wpi but by a conventional PCR, which is a less time consuming method, and amplifying a 292 bp fragment of the ITS2 gene. Ayaz, Ullah, AbdEl‐Salam, Shams, and Niaz (2014) compared the prevalence of *F. hepatica* in cattle and buffaloes using the FEC and a PCR method; authors showed the higher sensitivity of the molecular technique. Additionally, to differentiate between species, *F. hepatica* and *F. gigantica*, a single‐step multiplex PCR was developed for simultaneous detection using faecal samples (Le et al., 2012); both species overlap in distribution in some countries of Africa and Asia and have similar egg morphology, making identification from faecal samples difficult.

One of the drawbacks of the PCR is that this technique is only available in specific laboratories because of the need for specialized equipment. There are also problems in reproducibility between laboratories, with published methods often not working in other diagnostic laboratories. For these reasons, loop‐mediated isothermal amplification (LAMP) has been investigated as an alternative to PCR. LAMP assay is a very specific, efficient and rapid gene amplification procedure in which the reaction can run at a constant temperature (Notomi, 2000). Martínez‐Valladares and Rojo‐Vázquez (2016) developed a LAMP assay to detect fluke DNA in faeces of sheep and compared the results with a conventional PCR. Detection of infection was confirmed during the first wpi by both techniques, and in naturally infected sheep, the sensitivity was slightly higher with the LAMP assay. In this study, the standard PCR took around 3 hr to obtain a result, comparing with 1 hr and 10 min for the LAMP assay. However, Arifin, Hoglund, and Novobilsky (2016), after using the conventional PCR and LAMP assay in sheep and cattle, found poor sensitivity compared with FEC and sandwich‐ELISA techniques. The main reason of these different results between studies could be due to the DNA extraction protocols. Indeed, in the study by Martínez‐Valladares and Rojo‐Vázquez (2016), samples were subjected to ethanol precipitation after the DNA extraction, following the protocol described by Robles‐Pérez et al. (2013), to concentrate and purify the samples.

With the exception of the LAMP assay, all the diagnostic tests described for fluke infection require laboratory facilities. This extends the time taken to get results back to farmers and also increases cost. Pen‐side tests are urgently required by the industry. The FAMACHA© chart is a low cost tool for determining anaemia status in ruminants and can be used as part of an integrated worm control programme. Selective treatment of animals based on anaemic status is important in preventing anthelmintic resistance (Reynecke, van Wyk, Gummow, Dorny, & Boomker, 2011). In a study carried out by Olah, van Wyk, Wall, and Morgan (2015), authors found a significant correlation between the FAMACHA© score in sheep and the presence of flukes in the liver (*r* = .54, *p* < .001). However, in cattle naturally infected with *F. gigantica* and paramphistomes, the sensitivity and specificity of the FAMACHA© are low (Dorny et al., 2011; Elelu, Ambali, Coles, & Eisler, 2016).

Martínez‐Sernández et al. (2011) developed a lateral flow test (SeroFluke) for the serodiagnosis of human fasciolosis. In comparison with an ELISA test (MM3‐SERO), the SeroFluke test showed excellent specificity and sensitivity and could be used with serum or whole blood samples.

### 5.1 Key questions and future directions

Accurate, quick and simple diagnosis of *Fasciola* spp infection would allow targeted treatment and more accurate prevalence surveys, but diagnosis at the moment relies on detection of eggs or *F. hepatica*‐specific antigen in faeces, or detection of antibody in serum or milk. All of these assays require some form of laboratory equipment and results are normally only available to farmers several days after the sample has been collected All these assays have some limitation, for example, they only detect patent or historic infection and importantly, delays in receiving results often means treatments are applied in the absence of diagnosis of infection. For effective diagnosis of fluke infection, individual animal diagnostic tests are urgently required, supported by decision trees to help farmers interpret their results and administer drugs or implement control programmes effectively. The results presented here, describing lateral flow tests and other methodologies such as LAMP, show that developing pen‐side tests is feasible and is a research priority. Effective, simple and cost‐effective diagnosis will enable farmers to treat animals in an informed and targeted manner, and will help the industry move away from the traditional repeated blanket treatment regimes that have led to widespread resistance to flukicides.

## 6 ANTHELMINTIC RESISTANCE

A number of flukicide drugs are licensed for use in sheep and cattle, including the benzimidazole derivative triclabendazole (TCBZ), albendazole, closantel and clorsulon. Unique amongst these drugs, TCBZ demonstrates high efficacy against both adult parasites and immature fluke as early as 2‐day post‐infection (Boray et al., 1983), whilst the other flukicides only target flukes from 6‐ to 14‐week post‐infection (Kelley et al., 2016). As a result, TCBZ has become the drug of choice, particularly for treating acute fasciolosis in sheep, but this overreliance on TCBZ has inevitably resulted in the emergence of TCBZ‐resistance (TCBZ‐R) in liver fluke populations. The threat TCBZ‐R poses to the future control of liver fluke infections was the focus of a recent review which summarizes the number of cases reported worldwide, since the first report of TCBZ‐R in Australia in 1995 (Kelley et al., 2016; Overend & Bowen, 1995). To date, there are 20 peer‐reviewed reports of TCBZ‐R on sheep farms within Europe (Table 2), plus a number of anecdotal reports of resistance, which raises the question how prevalent is TCBZ‐R in Europe? It is not clear if reports of drug failure are recorded through veterinary medicine surveillance schemes and if so, if these data are available in the public domain? This information is needed to provide evidence for the widely held belief that resistance is widespread across Northern Europe and to give a clear picture of the TCBZ‐R status at an individual farm level, to ensure the most effective control measures are employed.

**Table 2:** Published Peer‐reviewed Reports of TCBZ‐R in Sheep in Europe (adapted from Kelley et al., 2016)

Year	Country or region	Number of farms	Ref
1998	Scotland	1	Mitchell, Maris, & Bonniwell (1998)
2000	The Netherlands^a^	1	Gaasenbeek, Moll, Cornelissen, Vellema, & Borgsteede (2001); Moll, Gaasenbeek, Vellema, & Borgsteede (2000)
2000	Wales	1	Thomas, Coles, & Duffus (2000)
2006	Spain	1	Alvarez‐Sanchez, Mainar‐Jaime, Perez‐Garcia, & Rojo‐Vazquez (2006)
2009	Republic of Ireland	1	Mooney, Good, Hanrahan, Mulcahy, & de Waal (2009)
2011	Scotland	1	Sargison & Scott (2011)
2012	Wales and Scotland	7	Daniel et al. (2012)
2012	Scotland	2	Gordon, Zadoks, Skuce, & Sargison (2012)
2015	Northern Ireland	5	Hanna et al. (2015)
Total no. farms on which reported		20	

a Reported in cattle as well as sheep.

One of the constraints to determining the full extent of TCBZ‐R is a lack of a quick and reliable diagnostic test to detect resistance in the field. All the studies shown in Table 2 used the faecal egg count reduction test (FECRT) method to identify resistance based on a <95% reduction in egg count 21‐day post TCBZ treatment. More recently the coproantigen reduction test has been favoured but still requires further validation in field studies (Kelley et al., 2016) and, as with the FECRT, it is time consuming and lacks the precision to directly detect drug resistance alleles as they emerge in parasite populations.

In vitro egg hatch assays have been used to detect resistance to albendazole and triclabendazole. Albendazole appears to have an ovicidal effect on *F. hepatica,* and egg hatch assays appear to discriminate between resistant and susceptible isolates (Canevari et al., 2013; Novobilský, Amaya Solis, Skarin, & Höglund, 2016; Robles‐Pérez, Martínez‐Pérez, Rojo‐Vázquez, & Martínez‐Valladares, 2014). In contrast, only one preliminary study suggested that TCBZ had an ovicidal effect (Fairweather et al., 2012), others showed that an egg hatch assay was not able to detect resistance to TCBZ in the field (Robles‐Pérez, Martínez‐Pérez, Rojo‐Vázquez, & Martínez‐Valladares, 2015) and in our experience, the amount of DMSO required to solubilize TCBZ and its metabolites is too toxic, hence if the appropriate controls are included, the assay cannot discriminate between resistant and susceptible field isolates of *F. hepatica* (Hodgkinson and Williams, unpublished observations).

Molecular tools to detect TCBZ‐R markers in field samples would provide precise data with which to target effective treatments and reduce the potential for TCBZ‐R parasites to move from farm to farm within infected livestock. Development of such a test relies on knowledge of the mode of action of TCBZ, which despite many years of study and the proposal of potential drug targets, for example, mictrotubule‐mediated activity and the adenylate cyclase pathway, remains unclear. Similarly, the mechanisms of TCBZ‐R are not known, although a number of candidates involved in altered drug uptake, efflux and/or metabolism have been proposed (Fairweather, 2009). This work has highlighted the importance of distinguishing between single nucleotide polymorphisms (SNPs) that are inherent in genetically diverse *F. hepatica* populations from those SNPs in genes conferring TCBZ‐R and deciphering the differential expression of proteins responsible for a resistance phenotype rather than those related to a general stress response (Kelley et al., 2016). These considerations have been the major driver behind a genome‐wide approach to mapping TCBZ‐R in genetically recombinant *F. hepatica* (Hodgkinson, Cwiklinski, Beesley, Paterson, & Williams, 2013). Our current lack of understanding of the mechanisms involved in TCBZ‐R raises several questions: Is there a single origin of TCBZ resistance or multiple origins and is a common pathway involved in the expression of a TCBZ‐R phenotype? Is TCBZ resistance a dominant or recessive trait? Is the same mechanism employed by both adult parasites and newly excysted juveniles (NEJ)? All of which comprise important knowledge gaps which limit our ability to mitigate the impact of TCBZ‐resistant liver fluke infections.

Alternative chemical options available to target TCBZ‐resistant fluke include treatment with clorsulon, nitroxynil, closantel, albendazole or oxyclozanide (dependent on the host species, (Coles & Stafford, 2001)). Whilst all these chemicals can control TCBZ‐resistant flukes none can kill the juvenile stage of the parasite and therefore have to be used more strategically than TCBZ has historically been used. Where possible it is important to avoid perceived TCBZ resistance from causing a change in on‐farm practice, where farmers rely heavily on alternative flukicides, such as closantel (McMahon et al., 2016). This is particularly pertinent given the recent report of the first case of closantel resistance in cattle in Sweden (Novobilský & Höglund, 2015); which in turn raises the question of how prevalent is closantel resistance in *F. hepatica*?

### 6.1 Key questions and future directions

If effective drug control is to be implemented, we require knowledge of the prevalence of flukicide resistance in Europe, in particular to TCBZ. We need to understand the genetic and molecular basis of resistance to flukicides in *F. hepatica* populations, especially to TCBZ and we require an appreciation of the factors that influence the emergence and spread of drug resistance alleles in liver fluke populations.

## 7 IMMUNITY TO *FASCIOLA* SPP

**Figure 1:**
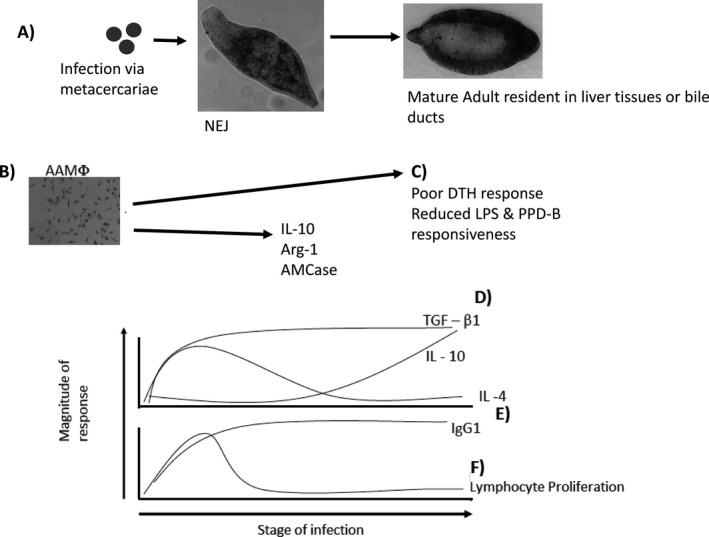
Immune responses to *Fasciola hepatica*; (a) as the life cycle begins within the mammalian host progressing from ingested metacercariae to NEJ to adult fluke multiple immunological events occur. (b) Alternatively activated macrophages (AAMφ) quickly emerge and are characterized by arginase‐1 and IL‐10 expression. These cells have been implicated in examples of host immunomodulation and bystander suppressive effects shown in (c). In parallel the adaptive response becomes detectable within 3–4 weeks post‐infection when both IL‐4 and IgG1 can be measured (e); however, only IgG1 levels are sustained. Simultaneously lymphocyte proliferation (f) is readily detected overlapping with NEJ presence in the intestine, the peak response is quickly reached and dissipates rapidly. IL‐4 (e) and TGF‐β (d) are thought to have T‐cell sources during infection and a plateau of TGF‐β appears to coincide with IL‐4 but only TGF‐β levels are maintained. Significantly, IL‐10 levels (d) are slower to rise in comparison with TGF‐β but remain during experimental infection. IgG1, lymphocyte proliferation and cytokine responses are correlated with the parasite burden indicating a strong role for antigenic load in driving these responses

The immune response to infection of ruminants with *F. hepatica* and *F. gigantica* is often simplified to a simple pattern that is seen during experimental infection of mice with *Schistosoma* spp. After an initial phase of antigen‐specific cellular proliferation and IFN‐γ synthesis [week 0–2], there is a shift to a Th2/type‐2 profile whereby production of IL‐4 and IL‐13 dominates and cellular responses become more variable and begin to decrease [week 2 – week 6–8] (Clery, Torgerson, & Mulchaly, 1996; Flynn & Mulcahy, 2008b). As infections become patent, there is a dramatic collapse in antigen‐specific cellular proliferation and the host cytokine profile becomes dominated by IL‐10 and TGF‐β. Superimposed on this general description are multiple waves of eosinophilia, alternative activation of macrophages and the progression, from the start of infection onwards, towards a dominant IgG1 antibody profile (summarized in Fig 1; (Flynn, Mulcahy, & Elsheikha, 2010)). However, the variety of host species that can be infected and the differences in the biology of *F. hepatica* and *F. gigantica* make deciphering results and finding commonalties difficult. Below we summarize immunological findings with regard to putative mechanisms of protective immunity, and their initiation and regulation.

### 7.1 Parasite recognition and innate effectors

Initial recognition of the metacercariae and NEJs takes place within the gastrointestinal tract meaning mucosal surveillance, and epithelial activation is potentially very important. In contrast to murine helminth models, there has been no work to date on the role of the canonical type 2 cytokines—IL‐25, IL‐33, TSLP—which have been shown to be crucial in the development of innate lymphoid cells (ILC2s) and subsequent CD4 T‐cell helper 2 (Th2) responses (Fallon et al., 2006; Humphreys, Xu, Hepworth, Liew, & Grencis, 2008; Neill et al., 2010; Oliphant et al., 2014; Taylor et al., 2009). This represents a major gap in our knowledge and potentially may yet influence the choice of adjuvants to be used in experimental vaccination if these cytokines are found to be important in directing the early host response against the NEJ.

Studies on the recognition of the NEJ have recently benefitted from the advances made in analysis of proteins and their carbohydrate residues. Garcia‐Campos et al. (2016) have found that the tegument of NEJs was primarily composed of oligomannose and core‐fucosylated truncated N‐glycans. The importance of these carbohydrate residues is re‐enforced by the finding that tegumental antigens (Teg) induce angeric T‐cells via dendritic cells (DCs) in a mannose receptor (MR)‐dependent fashion (Aldridge & O'Neill, 2016). A second study from the same group, however, demonstrated that despite the role of the mannose receptor binding components in Teg not all of its effects were MR‐dependent (Ravidà et al., 2016). Indeed the effects of Teg on DCs are known to TLR4‐independent (Hamilton et al., 2009), while those of excretory/secretory (ES) antigen on the same cell type is partially TLR4‐dependent (Dowling et al., 2010). These results corroborate previous studies, namely that ES antigens rely partially on both carbohydrates and TLRs to signal in bovine macrophages (Flynn & Mulcahy, 2008a).

While the recognition of invading NEJs is still under investigation we now understand that *F. hepatica* has a profound influence on a range of innate effector cells. One key mechanism that is becoming apparent is that host dendritic cells are targeted by a variety of antigenic fractions, or specific parasite proteins, that modulate the DC phenotype to suppress maturation (Dowling et al., 2010; Hamilton et al., 2009) and generate tolerogenic effects (Falcon et al., 2010) that were subsequently shown to suppress autoimmunity (Carranza et al., 2012). Specific mediators of these effects have been described including a Knutz‐like molecule (Falcón et al., 2014)and tegumental gylcans (Aldridge & O'Neill, 2016). Despite this, there is some suggestion that a component of *Fasciola* can direct a Th1‐like response through DC instruction and this could result in protection (Falcón, Carranza, Aoki, Motrán, & Cervi, 2011; Noya et al., 2015).

Similar effects have recently been reported in murine mast cells (Van Milligen, Cornelissen, Hendriks, Gaasenbeek, & Bokhout, 1998; Vukman et al., 2013), but the functional effect of mast cells has yet to be definitively shown as there is no evidence they are protective in rats (Van Milligen et al., 1998) (Van Milligen et al., 1998). Likewise in bovine infection there is little evidence mast cells increase in number or % following challenge with *F. hepatica* (Bossaert, Jacquinet, Saunders, Farnir, & Losson, 2000; McCole, Doherty, Torgerson, & Baird, 1998) but in the case of *F. gigantica* infection of buffaloes histological findings report increased mast cell numbers within the liver (Molina & Skerratt, 2005). No direct studies have addressed the role of basophils in *Fasciola* infection, but there is a brief spike in basophil number in circulation after infection (Poitou, Baeza, & Boulard, 1993). Given that there is a clear role for basophils as major IL‐4 secretors in response to protease‐type antigens, for example papain, during the initiation of the CD4 Th2 response (Sokol, Barton, Farr, & Medzhitov, 2007) and given the abundance of proteases in *F. hepatica* this is pertinent point for investigation. A study of the human neutrophil response found that patients with acute disease, harboured neutrophils with greater phagocytic function compared to those in the chronic stage of infection (Osman, Rashwan, & Farag, 1995). Similarly, a study of PMNs from infected goats found that infected animals had a poor phagocytic response compared to controls and this was also correlated with fluke burdens (Martínez‐Moreno et al., 2000). A defined role for neutrophils in antibody dependent cell cytotoxicity (ADCC), or other protective mechanisms, remains to be demonstrated in fluke‐infected cattle.

Eosinophils and macrophages have been shown to mediate ADCC against *F. hepatica* in rats (Van Milligen et al., 1998) and bovine macrophages ex vivo, can kill NEJs (Duffus, Thorne, & Oliver, 1980) although whether this killing is entirely antibody mediated is questionable (Glauert, Lammas, & Duffus, 1985). Indeed cathepsin L1 is known to cleave NEJ‐bound antibody during this process (Carmona, Dowd, Smith, & Dalton, 1993). In cattle and sheep, *F. hepatica* induces a liver and blood eosinophilia and *F. gigantica* infection of sheep gives the same profile (Chauvin, Moreau, & Boulard, 2001; Zhang et al., 2005). Paradoxically under the cover of vaccination calves and goats showing protection had reduced eosinophil counts (Wedrychowicz et al., 2007; Zafra, Pérez‐Écija, Buffoni, Moreno et al., 2013). This is further complicated by findings using Indonesian thin tailed (ITT) sheep who display resistance to *F. gigantica* but not *F. hepatica*. (Piedrafita et al., 2007)) demonstrated an ex vivo role for ADCC by eosinophils in killing *F. gigantica* but not *F. hepatica,* however, (Pleasance, Raadsma et al., 2011) showed that peripheral eosinophilia was not related to resistance to *F. gigantica*. These findings raise the prospect that eosinophils act only within the gut or peritoneal cavity, but not the liver of some host species in an infection specific manner. Macrophages have also been shown to mediate ADCC and again paradoxically a reduction in their alternative activation profile is also known to be linked to protection in calves during experimental vaccination (Golden et al., 2010), suggesting that ADCC mediated by macrophages is nitric oxide mediated, induced in a Th1 cytokine environment and potentially relying on IgG2a. Whether these effector cells, particularly macrophages and eosinophils, are targets of *F. hepatica* immunmodulation, because they are key mediators of protection, has yet to be fully understood.

### 7.2 Adaptive responses

The kinetics of B‐cells in both infected and vaccinated animals would suggest that they play a role in providing protection. Chung, Bae, Yun, Yang, and Kong (2012) noted an accumulation of splenic CD19^+^ B‐cells post‐infection in mice. Zafra et al. (2010) showed an accumulation of IgG^+^ cells in the cortex of the hepatic lymph node and liver that increased under vaccination. Likewise a similar infiltration of plasma cells in the livers of water buffalo and cattle is seen during *F. gigantica* infection (Molina & Skerratt, 2005). In sheep infected with *F. hepatica* there was an 11% increase in the number of B‐cells recruited into ovine hepatic lymph nodes (Meeusen, Lee, Rickard, & Brandon, 1995). The dynamics of antibody secretion are relatively straightforward with most experimentally infected animals seroconverting within 4 weeks of exposure (Flynn & Mulcahy, 2008c). In rats, a general increase in IgG1, IgG2a and IgE was seen throughout the course of a 10 week infection and only IgM was noted to decline (Poitou et al., 1993), whereas over a longer period of time, 7–12 weeks, only IgG1 and IgG2a were found to be maintained at levels above baseline (Gironès et al., 2007). In cattle, the balance of isotypes favours IgG1 over IgG2 and in both cattle and sheep IgG2 is linked with the expression of resistance or protection against infection (Golden et al., 2010; Pleasance, Wiedosari, Raadsma, Meeusen, & Piedrafita, 2011). Mechanistically IgG2a is known to be responsible along with eosinophils in mediating ADCC in rats (Van Milligen et al., 1998), while in cattle, experimental vaccination achieving protection relies on higher IgG2 (Mulcahy, 1998). Recently Fu et al. (2016) showed that IL12 and IL18 transcriptions are inhibited in sheep infected with *F. hepatica*, leading to changes in CD40 which are essential to B‐cell proliferation and class switching, suggesting one possible mechanism for the polarization of the IgG response in infected animals.

Studies of the adaptive cellular response have focused predominately on CD4 T‐cells, but some studies have indicated the presence of hepatic NK cells in rats producing IFN‐γ (Tliba, Chauvin, Le Vern, Boulard, & Sibille, 2002). The antigen‐specific recall response is known to be dominated by IFN‐γ early in ruminants (Clery et al., 1996; Flynn & Mulcahy, 2008b) and rodents (O'Neill et al., 2000). The shift to an IL‐4/Th2 dominant response is also known to occur in cattle and mice, and this is known to be more pronounced in the mesenteric and hepatic lymph nodes (Flynn & Mulcahy, 2008b; Tliba et al., 2002). Insights into the chronic state or suppressed cellular responses have recently seen conflicting findings that Foxp3 is upregulated (Walsh, Brady, Finlay, Boon, & Mills, 2009)but that anergic T‐cells, characterized by PD‐1, might have a bigger role to play. Recent evidence in ruminants suggest that γδ T‐cells, not CD4 T‐cells, are the major expresser of Foxp3 (Guzman et al., 2014)and that immune checkpoint molecules such as PD‐1, CTLA‐4 and LAG1 might have greater roles to play in non‐responsive CD4 T‐cells during chronic infection (Ikebuchi et al., 2014). What has become apparent from a number of recent transcriptomic studies is that the *il12*,* tnf*, and *ifng* complex is downregulated in ovine PBMCs as infection progresses (Fu et al., 2016), whilst within the liver, genes mediating Th2 cellular responses were upregulated (Alvarez Rojas et al., 2014).

#### 7.2.1 Key questions and future directions

While great progress has been made in recent years much is yet to be understood about the fundamental nature of the host‐parasite relationship and a number of areas need to be addressed. Firstly, the vast bulk of our knowledge draws on experimental infections; well‐controlled experiments studying the dynamics of host immunity in field conditions are desperately needed. These will help to validate or discount experimental data and also provide vital baseline information to feed into vaccination trials. The interpretation of these field data will need to be approached cautiously to carefully model the vastly more confounding variables influencing such experiments. Secondly, what role do the canonical type‐2 cytokines have on the initiation of the immune response? The consensus from murine models of infection would suggest that these cytokines in conjunction with the cells they elicit, the ILC2s, are pivotal in orchestrating immunity. Finally, determining the mechanisms of parasite killing is key to developing effective vaccines. Clearly there is a role for ADCC; however, the site of its action and the host cells involved remain to be further elucidated.

## 8 VACCINE DEVELOPMENT

The current control of *F. hepatica* relies primarily on the use of anthelmintic drugs. In view of a changing climate, increasing prevalence of infection and resistance to the limited number of flukicides available, there is an urgent need for alternative control methods. During the last two decades, vaccines have been considered a promising and economically viable alternative strategy for the control of fasciolosis in livestock (McManus & Dalton, 2006; Molina‐Hernández et al., 2015; Toet, Piedrafita, & Spithill, 2014; Yap & Smooker, 2016). Fluke vaccine studies have been conducted in laboratory animals such as rats, mice and rabbits (reviewed by (Meemon & Sobhon, 2016)) and in ruminants (cattle, sheep and goats). Vaccine trials in rodent models are much cheaper than conducting trials in ruminants, and they are useful to study the mechanism of the immune response in protected and non‐protected animals, but the levels of protection recorded in animal models, particularly in the rat, are difficult to replicate in livestock species.

A crucial question for a successful commercial fluke vaccine is the level of protection required to increase livestock production. As production losses are observed in sheep showing 30–54 flukes (Dargie, 1987), in herds with low and high fluke burdens a protection of 50% and 80%, respectively, would be sufficient (Toet et al., 2014). Recent modelling studies have reported that a vaccine inducing a reduction in fluke burden of 43%, but which protects 90% of the herd and lasts for a full season, would have an important impact on the control of disease (Turner et al., 2016). Many prototype vaccines, although they have a variable effect on fluke burden, have a significant effect on egg output and egg viability. Hence more attention is needed to evaluate these effects in vaccine trials. As *F. hepatica* causes important hepatic damage in sheep, particularly during the migratory phase, even without significant fluke reduction some trial vaccines induced less hepatic damage and higher weight gain than unvaccinated controls (Zafra, Pérez‐Écija, Buffoni, Pacheco et al., 2013). This suggests that reduction hepatic damage will have an impact in reducing production losses.

A better understanding of the mechanism of the protective response against *Fasciola* spp in ruminants is vital to fully understand how best to develop and deliver vaccines (Molina‐Hernández et al., 2015). In vaccinated cattle, a Th1 response with high titres of IgG2 and low titres of IgG1 has been correlated with protection (Mulcahy et al., 1999). More recent trials in cattle (Golden et al., 2010), sheep (Maggioli et al., 2011)and goats (Villa‐Mancera, Reynoso‐Palomar, Utrera‐Quintana, & Carreón‐Luna, 2013) found high levels of IgG2 and IgG1 associated with protection, possibly indicating a mixed Th1/Th2 response. As described in Section 7, *Fasciola* spp. has the capacity to modulate the host response facilitating parasite survival during both acute and chronic stages of infection. Alternative activation of macrophages, a skewed Th2 response and expansion of regulatory T cells and apoptosis of effector cells (Dalton, Robinson, Mulcahy, O'Neill, & Donnelly, 2013; Escamilla, Bautista et al., 2016; Escamilla, Zafra et al., 2016; Fu et al., 2016) have been reported in laboratory animals and ruminants. Cathepsins (CL), peroxiredoxin (Prx) and helminth defence molecules (HDM) are thought to be involved in several immunomodulatory mechanisms (Dalton et al., 2013). The inclusion of immunomodulatory molecules in vaccines formulated with appropriate adjuvants to enhance Th1 responses may help to reduce the effects of parasite immunomodulation thus increasing vaccine efficacy. A protective host response has been described in the early stages of infection affecting the peritoneal or early hepatic migratory stages of the parasite; however, recent vaccine trials against *F. hepatica* in rats have reported that, at least in part, protective responses occur in the biliary compartment (Wesołowska et al., 2017). These are interesting data as an effective response against mature flukes would lower egg output and egg viability, which in turn, would reduce transmission.

The duration of protective immunity is important for the commercial success of a vaccine, but little information on the longevity of the protective response is available from experimental trials. Modelling studies have shown that protection that lasts a whole grazing season is required for a vaccine to be commercially viable (Turner et al., 2016). The age of protected animals is also relevant as lambs and calves are put out onto pasture between one and 2 months of age. Hence for a vaccine to be viable, it should be effective in young animals as well as older stock and to date most of vaccine trials have been focused on sheep and cattle with more than 4 months of age.

A variety of native purified antigens have been used in vaccine trials in cattle and sheep, including native fatty acid binding proteins (FABP) induced 55% protection in cattle against *F. hepatica*; native cathepsin L1 (CL1) induced 42%–69% protection in cattle and 34% protection in sheep; native glutathione S transferase (GST) induced 57% worm reduction in sheep and 0%–69% protection in cattle. Native leucine aminopeptidase (LAP) induced 89% protection in sheep (reviewed by (Toet et al., 2014; Yap & Smooker, 2016)). As native fluke antigens are not viable for a commercial vaccine, research has focused on developing individual recombinant antigens. However, numerous trials have shown no significant or discrete protection (Toet et al., 2014; Yap & Smooker, 2016). Some trials using recombinant antigens have shown very high protection, for example, a trial using recombinant *Schistosoma mansoni* 14 antigen (rSm14) in RIBI adjuvant, induced 98.5% protection in (Almeida et al., 2003), although the groups were small (*n* = 4), and protection was not confirmed in subsequent trials in goats (Mendes et al., 2010). A second study used rLAP from *F. hepatica* given in different adjuvants in sheep, and led to a reduction in fluke burden of 74% and 86% with adjuvac 50 and alum adjuvant, respectively (Maggioli et al., 2011). Using CL1/CL2 mimitopes, Villa‐Mancera et al. (2008) reported 47% protection in sheep, and CL1 mimitopes on their own led to protection of 51% in sheep (Villa‐Mancera & Méndez‐Mendoza, 2012) and 46%–79% in goats (Villa‐Mancera et al., 2013). These promising results in sheep and goats have yet to be validated in subsequent trials and in field trials.

As for several other helminth vaccines, it is recognized that fluke vaccines based on single molecules are probably unrealistic, hence different antigen combinations have been evaluated. To date, most combinations have been based on native rather than recombinant antigens, for example, CL1+ Haemoglobin (Hb), CL2+ Hb and CL1+ CL2. With these combinations, protection ranged from 0% in sheep to 72% in cattle (Dalton, McGonigle, Rolph, & Andrews, 1996; Mulcahy et al., 1999) whilst native CL1+ CL2+ LAP induced a reduction of 79% in worm burden in sheep (Piacenza, Acosta, Basmadjian, & Carmona, 1998). More recent studies using recombinant proteins showed promising results: in cattle immunized with recombinant cathepsin L1 (CL1) a significant reduction in fluke burden of 48% was found (Golden et al., 2010); and in buffalo, recombinant FABP and GST induced a 35% reduction in *F. gigantica* (Kumar et al., 2011). A combination of several immunomodulatory and biologically relevant parasite molecules with the appropriate adjuvant and/or delivery system to drive a Th1 or a mixed Th1/Th2 response may enhance vaccine protection. Antigen competition should also be taken into account when using vaccines with multiple antigen combinations.

### 8.1 Key questions and future directions

The significant levels of protection, in terms of a reduction in fluke burden, faecal egg output and egg viability found in trials using combinations of antigens, in cattle, sheep and goats, suggest a fluke vaccine is a feasible and viable method for the control of fasciolosis in livestock that will lessen anthelmintic use and slow the spread resistance. Nevertheless, much more work is needed to: (i) confirm the reproducibility of those trials under both experimental and field conditions; (ii) improve our understanding of protective and non‐protective immune mechanisms in ruminants and identify those parasite molecules involved in immunomodulation; (iii) investigate with the most appropriate antigen/adjuvant combinations for sheep and cattle to improve vaccine efficacy; (iv) better understand the duration of immunity, how animal age affects vaccine success and to quantify the effect of reduced egg number and viability on transmission of the parasite.

## 9 CONCLUSIONS

Liver fluke is a common parasite that affects the productivity and welfare of cattle and sheep. Control is confounded by the lack of low cost, accurate, animal‐side diagnostics, issues surrounding use of drugs in milking cattle, anthelmintic resistance and climate change that will significantly alter the epidemiology and transmission of the parasite over the coming decades. Research is urgently needed into many areas of fluke biology, and control that this review has sought to highlight. The availability of an annotated, whole‐genome map of *F. hepatica* (Cwiklinski et al., 2015) will accelerate developments in many of these areas. Better advice on control of the parasite at the farm level is also imperative and will depend on our growing understanding of the complex interactions between environment, ecosystems, snail host, wildlife hosts and farmed animals.
